# Correspondence

**Published:** 1866-06

**Authors:** James P. White

**Affiliations:** Alhambra, Grenada, Spain


### Correspondence.

---

ALHAMBRA, GRENADA, SPAIN, April 1, 1866.

*To the Editor of the Buffalo Medical and Surgical Journal:*

My Dear Doctor:—We left Madrid on the 16th of March. The weather, even in that city, elevated more than 2300 feet above the level of the sea, and in nearly the same latitude as Buffalo, was warm and pleasant. Proceeding south to Alcozar and Cordova, we found immense groves of orange-trees in blossom, bearing green and well ripened fruit at the same time, and often upon the same tree. In Cordova there remains but the debris of a university, at one time so celebrated as to attract students from all Europe, and whose medical faculty was composed of the most celebrated men of their day. It still keeps up an organization, but it is in its senility, a shadow only of its former greatness. They have an immense "Corso de Toros," or amphitheater, for their favorite amusement in this city, and I witnessed one of these barbarous entertainments. Four bulls were killed, two horses being gored to death by these infuriated animals before they were conquered. Unfortunately the riders of these poor slaughtered horses escaped with little injury. Perhaps I am wrong in so wishing, but I could not resist the desire that the brutal men engaged in this inhuman sport! might suffer rather than their dumb victims, in the hope that in this way the horrible practice might be reformed. It is a barbarous practice to which the Spanish cling with great pertinacity, but it is to be hoped that the process of opening up the country by railroads, which is now going on, and the consequent introduction of large numbers of travelers will ere long dissipate this relic of a dark and bloody age. But I will not detain you with a dissertation upon this disagreeable subject, or with a particular description of the tournament which I witnessed.

In Cadiz there is also an old university foundation with a medical department, which possesses considerable vitality. The faculty of medicine consists of men of ability, who lecture annually to respectable numbers of young barbers and other students. The lecture rooms are all small, the students being divided into small classes, and the teacher delivers his lecture in a sitting posture, occupying in some instances a sort of pulpit, somewhat elevated above his class. By the professor of surgery I was shown a very remarkable case of nevus maternus. It occupied the mouth, tongue and left cheek and lower lip. The tongue was immense, as large as the tongue of an ox, and the whole organ nearly back to the epiglottis, black with distended congeries of vessels of which it seemed composed. They were then applying electro-galvanism by means of small points which were inserted into the distended mass of vessels, and then the battery applied with as much power as the patient could bear. The professor informed me that it had been applied six times to the cheek, and they proposed next to insert the points into the tongue. The operater himself and all who had witnessed the effect of the cauterization upon the cheek and lips were sanguine of ultimate success; and there was a large shrivelled eschar upon the surface to which the applications had been made, which demonstrated that their expectations were not without good foundation. Being promised the result of the treatment in this interesting case it shall be transmitted for the benefit of your readers when received.

In the museum of this institution, which is large and well arranged, is exhibited a well preserved specimen of an adult male chest, in which the stomach is seen above the diaphragm and in the position usually occupied by the heart. The heart was crowded quite over to the right side. No other mal-formation was observed in this individual. He lived to the age of twenty-four years and died of a wound received in a row. The deformity was discovered only after he was placed upon the dissecting table.

There is a large and well regulated hospital connected with the school in Cadiz, affording ample opportunity for clinical instruction. This hospital liberally admits sailors who may be sick or wounded in this port, from all parts of the world. An Englishman was then in hospital, who, in a fit of mania portu, had cut his throat from ear to ear, nearly severing the trochea. The wound was nicely dressed, and the poor fellow pale from the loss of blood now quite sane, wrote upon a slate in answer to my inquiries, that he was respectably connected, was kindly treated in the hospital, and as his friends were not aware of his disgrace he was coming to America to *reform*, in case of his recovery.

Nothing of great interest in a medical point of view came under observation between Cadiz and Malaga. The last mentioned city is situated on the northern shore of the Mediterranean, about one hundred miles east of Gibralter. It lies in an amphitheater, surrounded by lofty hills upon the east and north, which extend well round to the west. It is thus well protected from the cold winds which prevail at certain periods from those directions. The prevailing winds are from the south during winter, thus passing over the sea, upon the borders of which the city is situated. The temperature of the Mediterranean at this point does not vary a single degree in the entire year. The number of days upon which rain falls during the year does not, as established by observations extending over many years, exceed thirty-eight. The temperature varies less than in any other known district, except Madurea, which has many more rainy days, and a much more humid climate throughout the year. The climate of Malaga is warm, less humid, and much less variable than the greatly celebrated Riverid or eastern slope, so to say, of the Mediterranean betwcen Nice and Genoa. Nice, Montone, and the other towns of the eastern coast have long been celebrated for their salubrity and continue to be favorite resorts for invalids threatened with pulmonary tuberculosis. Do not understand me as doubting the advantages of a winter residence at Nice, rather than in England or the Northern States of America. My belief, however, is, that in all the essentials of a climate favorable to recovery of pulmonary affections Malaga far surpasses them. It has fewer days of rain, hence the invalid may take exercise in the open air, which I consider of the utmost importance by just so many days more, instead of being shut up in-doors. Malaga has a higher average range of thermometer with less variation; and a higher average range of the hygrometer than Nice or any of the Italian cities.

Within fifty or sixty miles of Malaga by ascending into the mountainous district of Granada, the same equable temperature can be found for the invalid during the hot summer months, when both Nice and Malaga become unsuitable residences. Nothing can be more attractive than the natural scenery about Grenada, and there is no place which to the tourist would possess greater interest than the Alhambra. Everything here would invite open air exercise, so essential to the phthisical invalid. Hotels are comfortable in Grenada, and horses can be hired at reasonable prices for rides into the surrounding mountains and Gipsey villages. Nice and Montone have at this moment better hotel accommodations than can be found in Malaga. The hotels in the latter place are very comfortable and less expensive than those of the eastern coast, but in every other respect the advantages are, in my opinion, greatly in favor of Spain. Malaga may now be reached by rail from the north, and I cannot doubt that it will ere long become a favorite winter resort for all with weak lungs or other diseases requiring a mild, uniform temperature, dry air and ample opportunities for out-door exercise.

But I will not detain you with what I confess has become with me a hobby, and therefore subscribe myself,

Truly yours, JAMES P. WHITE.

